# RNA-Seq Analysis of the Host Response to *Staphylococcus aureus* Skin and Soft Tissue Infection in a Mouse Model

**DOI:** 10.1371/journal.pone.0124877

**Published:** 2015-04-22

**Authors:** Rebecca A. Brady, Vincent M. Bruno, Drusilla L. Burns

**Affiliations:** 1 Division of Bacterial, Parasitic, and Allergenic Products, Center for Biologics Evaluation and Research, FDA, Silver Spring, Maryland, United States of America; 2 Institute for Genome Sciences, University of Maryland Baltimore, Baltimore, Maryland, United States of America; Public Health Research Institute at RBHS, UNITED STATES

## Abstract

*Staphylococcus aureus* is a leading cause of skin and soft tissue infections (SSTI), which are primarily self-limiting. We conducted a comprehensive analysis of the host transcriptome during a *S*. *aureus* SSTI to provide insight on the protective mechanisms that thwart these infections. We utilized a murine SSTI model in which one ear is epicutaneously challenged while the other is not. We then harvested these infected and uninfected ears, as well as ears from naïve mice, at one, four, and seven days post-challenge, and performed RNA sequencing (RNA-seq) using the Illumina platform. RNA-seq data demonstrated a robust response at the site of infection. Comparison of gene expression profiles between infected ears and the non-infected ears of challenged mice defined the local response to infection, while comparisons of expression profiles of non-infected ears from challenged mice to ears of naïve mice revealed changes in gene expression levels away from the site indicative of a systemic response. Over 1000 genes exhibited increased expression locally at all tested time points. The local response was more robust than the systemic response. Through evaluation of the RNA-seq data using the Upstream Regulator Analytic as part of the Ingenuity Pathway Analysis software package, we found that changes in the activation and inhibition of regulatory pathways happen first locally, and lag behind systemically. The activated pathways are highly similar at all three time points during SSTI, suggesting a stable global response over time. Transcript increases and pathway activation involve pro- and anti-inflammatory mediators, chemotaxis, cell signaling, keratins, and TH1/TH17 cytokines. Transcript decreases and pathway inhibition demonstrate that metabolic genes and anti-inflammatory pathways are repressed. These data provide insight on the host responses that may aid in resolution of this self-limited *S*. *aureus* infection, and may shed light on potential immune correlates of protection for staphylococcal SSTI.

## Introduction

*Staphylococcus aureus* is a normal colonizer of the human nose that is found in almost one-third of the population [1]. *S*. *aureus* features a broad and highly redundant repertoire of virulence factors that allow it to colonize and damage the host, as well as evade host immune responses and cause a variety of diseases in all areas of the body. As a result, this bacterium is a leading cause of both nosocomial and community-acquired infections in the United States [2,3]. In recent years, *S*. *aureus* infections have become more prevalent in the community, infecting patients with no predisposing risk factors [3].

Most community-acquired *S*. *aureus* infections occur in the skin and soft tissue and include boils, impetigo, cellulitis, folliculitis, and abscesses, often caused by isolates of the USA300 sub-type [4]. While these infections are largely self-limiting in nature, severe invasive illness can result [5]. Therefore, an understanding of the protective immune response in the skin is important in order to elucidate potential new ways of combatting *S*. *aureus* while it remains localized in this area.

Investigators are beginning to understand some facets of the host response to *S*. *aureus* SSTI. The skin itself acts as an immunologic barrier to infection, with its surface maintaining an unsuitable temperature and pH for bacterial growth [6]. Antimicrobial peptides such as β-defensin, as well as skin commensals such as *Staphylococcus epidermidis* and their secreted products (e.g., phenol-soluble modulins [7]) also work to inhibit *S*. *aureus* growth [6]. If an infection does take hold, neutrophil recruitment to the site of infection has been demonstrated as crucial for subsequent protection [8], mediated by pro-inflammatory cytokines such as IL-1β [9,10] and by IL-17, the product of a TH17 adaptive response [11].

Much still remains unknown about the host response to *S*. *aureus* SSTI, and, specifically, what correlates with resolution of these infections. While microarray analyses have been performed examining the murine gene expression profile in *S*. *aureus*-infected skin [9], limitations to microarray studies can lead to incomplete results. Next generation sequencing, such as RNA-seq, can provide greater sensitivity and eliminates issues with hybridization and nonspecific detection [12]. Therefore, in this study we utilized RNA-seq to generate transcriptional profiles of skin from the ears of mice infected with *S*. *aureus* sub-type USA300 using an epicutaneous infection model [13,14]. We compared the differential gene expression of skin from infected ears to that from uninfected ears from the same challenged mice over time in order to evaluate, for the first time, the local response to infection directly at the site of SSTI. We also compared the non-infected ears from challenged mice to naïve mice in order to elucidate the systemic response to SSTI challenge. The data generated provide further insight into the host response to *S*. *aureus* over the course of a self-limited infection, both locally and systemically, which may be useful in determining potentially novel pathways that are important for clearance of the pathogen.

## Materials and Methods

### Ethics Statement

For all animal studies, protocols were reviewed and approved by the Institutional Animal Care and Use Committees (IACUC) of the Center for Biologics Evaluation and Research (Silver Spring, MD; permit number 2010–04). All surgeries were performed under ketamine/xylazine anesthesia, and all efforts were made to minimize animal suffering. Animals were sacrificed at the time points indicated below using CO_2_ inhalation.

### Strains, mice, and reagents

*S*. *aureus* USA300 strain SAP149 [15] was used to infect mice. Ten week old female BALB/c mice were obtained from NCI (Frederick, MD). All animal experiments were approved by the CBER Institutional Animal Care and Use Committee. Unless indicated, all reagents were obtained from ThermoFisher Scientific (Rockville, MD).

### Epicutaneous *S*. *aureus* infection

The epicutaneous *S*. *aureus* challenge was performed as described by Prabhakara *et al*. [13]. Briefly, tryptic soy broth (TSB) was inoculated 1:50 with an overnight culture of SAP149, and then grown at 37°C with shaking until an absorbance at 600 nm (A_600_) of 0.8 was reached. The culture was then centrifuged at 3000 × *g* for 15 minutes and the pellet was resuspended in sterile PBS. The bacteria were then counted using a Petroff Hauser cell counter (Hausser Scientific, Horsham, PA). The bacteria were centrifuged again and then resuspended in PBS to a density of 1x10^11^ CFU/mL.

Mice were anesthetized using 2 mg Ketamine (Ketaject, Phoenix Pharmaceutical, St. Joseph, MO) and 0.1 mg Xylazine (AnaSed, Akorn, Decatur, IL) and the left ears were cleaned with 70% ethanol. The left ears were then pricked 10 times with a Morrow Brown allergy test needle (Morrow Brown Allergy Diagnostics, Oakhurst, NJ) containing a 10 μL drop of the *S*. *aureus* suspension. After infection, animals were monitored daily. Because the infection causes minimal distress, no analgesics were given. At each subsequent time point, subsets of mice were euthanized and ears were excised with scissors. Each ear was homogenized immediately in 5 mL of TRIzol (Life Technologies, Grand Island, NY). The homogenized ears were placed at -80°C until RNA isolation. For Western blotting, ears were excised and placed into lysis buffer on ice.

### RNA isolation

Frozen ear homogenates were thawed on ice, and the 5 mL TRIzol/homogenate mixture was then added to an additional 15 mL of TRIzol in a 50 mL conical tube. The tubes were incubated at room temperature for five minutes, and then 5 mL of chloroform was added and the tubes were shaken vigorously for 15 seconds. The tubes were incubated at room temperature for an additional three minutes, and then centrifuged at 5000 × *g* for 30 minutes at 4°C. The upper aqueous phase was retained in a new 50 mL conical tube and 10 mL of isopropanol was added and vortexed. The tubes were incubated at room temperature for 10 minutes and then centrifuged again at 5000 × *g*, 4°C, for one hour. The supernatant was decanted and the pellet was resuspended in ice cold 75% ethanol and placed at -20°C overnight. The next day, the precipitated RNA was isolated by centrifugation at 5000 × *g* at 4°C for one hour, the supernatant was removed, and the pellets were dried in a laminar flow hood for 20 minutes with the tubes inverted. The pellets were then resuspended in diethylpyrocarbonate (DEPC) treated water (Life Technologies) and quantified spectrophotometrically (NanoVue, GE Life Sciences, Pittsburgh, PA). The RNA was then alcohol precipitated a second time, resuspended again in DEPC water, and re-quantified spectrophotometrically prior to RNA-seq analysis. RNA integrity was evaluated both by agarose gel electrophoresis and BioAnalyzer analysis.

### RNA-seq and gene expression analysis

All RNA-seq libraries (non-strand-specific, paired end) were prepared with the TruSeq RNA Sample Prep kit (Illumina, San Diego, CA). The total RNA samples isolated from the SSTI infections were subject to poly(A) enrichment as part of the TruSeq protocol, and 100 nt of sequence was determined from both ends of each cDNA fragment using the HiSeq platform (Illumina) per the manufacturer’s protocol. Sequencing reads were annotated and aligned to the UCSC BALB/c mouse reference genome using TopHat [16]. For analysis of the infection data, the alignment files from TopHat were used to generate read counts for each gene and a statistical analysis of differential gene expression was performed using the DESeq package from Bioconductor [17]. We made comparisons between naïve uninfected mice and unchallenged ears from infected mice, as well as between challenged and unchallenged ears from the same infected mouse, at one, four, and seven days post-challenge. Three naïve and three infected mice were evaluated at each time point. A gene was considered differentially expressed if the false discovery rate (FDR) for differential expression was less than 0.01 and the fold change was at least two-fold (Log_2_ fold change (LFC) of ⩾1).

### Selection of top differentially expressed genes

All significantly differentially expressed genes for all time points are presented in the S1 Table. For simplicity of analysis, we chose to present only the 50 genes demonstrating the greatest increase or decrease in transcript levels from each time point within the manuscript. Under most tested conditions, we note that a proportion of differentially expressed genes show an up-regulation level of “*inf*”. These values occur when readings from the comparator ear (the non-infected ear from challenged mice when examining the local response, or the naïve ear from unchallenged mice when examining the systemic response) are below the limit of detection (i.e. no reads mapped to this gene in these samples). However, it is unrealistic biologically that the readings are truly zero. Because any level of reads in the test ear (i.e., the infected ear when examining the local response, or the uninfected ear from challenged mice when evaluating the systemic response) will then give a LFC value of *inf*, the magnitude of the actual difference is lost; we are unable to differentiate whether this difference is a few reads or thousands of reads. Therefore, for genes with LFC values of *inf*, we looked at the raw read data to determine the actual read levels in the test ear. In order to report a gene with a LFC of *inf* as a top differentially expressed gene (Tables 1–4), we chose to include only those genes that had a read level in the test ear that was higher than the smallest number of reads seen for any gene within the top 50 list that had a LFC of a numerical value. Alternatively, we also included genes with lower read values than this minimum, but a LFC of *inf* at multiple time points. We hypothesize that low read numbers consistently detected in test ears with undetectable reads in the comparator ears in different cohorts of mice, at multiple time points, likely reflect a real result. We also included genes that had a LFC of *inf* at one time point, but a significant differential expression with a numerical LFC at another time point. Through these criteria, we believe we have narrowed the genes with a LFC of *inf* to most likely reflect meaningful expression changes.

**Table 1:** Top Genes with Significantly Increased Transcript Levels at the Site of SSTI^1^.

Symbol	Gene Name	Function^2^	LFC^3^		
			Day 1	Day 4	Day 7
***Immune Function***					
Nlrp12	NLR family, pyrin domain containing 12	Suppression of inflammation	Inf	Inf	Inf
Pdyn	Prodynorphin	Pain/Stress perception; anti-apoptotic	Inf	Inf	Inf
Slc32a1	solute carrier family 32 (GABA vesicular transporter), member 1	GABA uptake into synaptic vesicles	Inf	Inf	6.27
Chst4	carbohydrate (chondroitin 6/keratan) sulfotransferase 4	L-selectin biosynthesis	Inf	Inf	5.49
IL-17a	interleukin 17A	Cytokine	5.13	Inf	2.88
Cxcl5	chemokine (C-X-C motif) ligand 5	Chemotactic for neutrophils	9.01	8.10	7.00
Cxcl3	chemokine (C-X-C motif) ligand 3	Chemotactic for neutrophils	8.97	6.87	6.78
Cxcl2	chemokine (C-X-C motif) ligand 2	Chemokine produced at sites of inflammation	8.73	6.49	6.45
Irg1	immunoresponsive gene 1	Suppression of inflammation	8.62	6.67	5.84
Ccl4	chemokine (C-C motif) ligand 4	Inflammatory chemokine	8.62	6.12	5.70
Trem1	triggering receptor expressed on myeloid cells 1	Inflammation	8.56	6.81	5.98
Csf3	colony stimulating factor 3 (granulocyte)	GCSF; cytokine involved in granulocyte production	8.36	6.59	6.49
Fpr1	formyl peptide receptor 1	Neutrophil activation	8.29	6.48	5.93
IL-1b	Interleukin 1 beta	Pro-inflammatory cytokine	8.08	6.36	5.87
Ccl3	chemokine (C-C motif) ligand 3	Inflammatory cytokine	8.02	5.85	5.75
Saa3	Serum amyloid A 3	Acute phase protein	7.79	8.02	6.34
IL-19	interleukin 19	Immunosuppressive during skin infection	7.73	7.21	6.33
Clec4e	C-type lectin domain family 4, member e	Pro-inflammatory receptor	7.71	6.15	5.46
IL-6	Interleukin 6	Lymphocyte differentiation; induces acute phase response	7.64	6.03	3.97
S100a9	S100 calcium binding protein A9 (calgranulin B)	Component of calprotectin	7.55	7.07	6.33
Reg3g	regenerating islet-derived 3 gamma	Antimicrobial	7.44	7.77	7.96
Olfm4	olfactomedin 4	Neutrophil granule protein; negative regulator of host immunity	7.43	7.45	5.19
IL-24	Interleukin 24	Cytokine	7.36	7.80	6.76
S100a8	S100 calcium binding protein A8	Component of calprotectin	7.35	7.38	6.41
Cxcr1	chemokine (C-X-C motif) receptor 1	IL8 receptor	7.05	5.80	4.33
Treml4	triggering receptor expressed on myeloid cells-like 4	Antigen presentation	6.95	5.24	5.93
Defb3	Defensin beta 3	Antimicrobial	6.19	7.57	6.09
Ly6g	lymphocyte antigen 6 complex, locus G	Signaling; neutrophil marker	5.88	5.07	3.42
IL-22	interleukin 22	Pro-inflammatory cytokine	4.19	7.17	4.37
Cxcl9	chemokine (C-X-C motif) ligand 9	Potentially involved in T cell trafficking	2.14	3.52	6.51
Defa-ps12	Alpha defensin, pseudogene 12	Pseudogene	—	Inf	Inf
Mcpt1	mast cell protease 1	Peptidase found in mast cell granules	—	Inf	Inf
Cmtm1	CKLF-like MARVEL transmembrane domain containing 1	Chemokine-like family	—	Inf	6.60
Cd5l	CD5-antigen like	Immune system regulation; inhibitor of apoptosis	—	4.29	Inf
Saa2	Serum amyloid A2	Acute phase protein	7.94	3.63	—
Cxcl15	Chemokine (C-X-C motif) ligand 15/IL8	Chemokine	Inf	—	Inf
Csf2	Colony stimulating factor 2	Cytokine involved in granulocyte production	8.37	—	—
Fcnb	ficolin B	Pattern Recognition Receptor	5.22	—	—
Gzmk	granzyme K	Serine protease; released from cytoplasmic granules of CTLs and NK cells	—	—	5.58
***Metabolism/Cell Proliferation/Regulation***					
Stfa1	Stefin A1	Cysteine protease inhibitor/Epidermal development	Inf	5.96	5.61
Drd2	dopamine receptor D2	Hormone regulation	4.44	5.99	Inf
Klk1b27	kallikrein 1-related peptidase b27	Peptidase	Inf	3.64	3.30
Fgf23	fibroblast growth factor 23	Regulator of phosphate homeostasis	8.78	8.01	5.42
Tdgf1	teratocarcinoma-derived growth factor 1	Growth factor	7.87	7.39	7.09
Mrgpra2b	MAS-related GPR, member A2B	Receptor	7.67	6.39	5.50
Stfa2l1	stefin A2 like 1	Thiol proteinase inhibitor	7.63	6.72	6.05
Mrgpra2a	MAS-related GPR, member A2A	Receptor	7.67	6.10	4.57
Tdgf1-ps1	teratocarcinoma-derived growth factor, pseudogene 1	Growth factor, pseudogene	7.61	7.21	6.15
Stfa2	Stefin A2	Thiol proteinase inhibitor	7.13	7.48	7.22
Abca13	ATP-binding cassette, sub-family A (ABC1), member 13	Transporter	6.08	4.39	4.33
Reg1	regenerating islet-derived 1	Islet cell regeneration	5.81	8.58	7.45
Uox	Urate oxidase	Convers uric acid to allantoin	4.84	7.60	7.13
Lhx1	LIM homeobox protein 1	Transcription factor	4.21	6.66	5.40
Cyp2j11	cytochrome P450, family 2, subfamily j, polypeptide 11	Arachidonic acid metabolism	Inf	Inf	—
Slc9c1	solute carrier family 9, subfamily C, member 1	Sodium-hydrogen exchanger; regulates intracellular pH of spermatozoa	3.76	Inf	—
Dynap	Dynactin associated protein	Regulation of cell proliferation	—	Inf	Inf
Cyp4a12a	cytochrome P450, family 4, subfamily a, polypeptide 12a	Arachidonic acid metabolism	—	Inf	Inf
Vmn1r210	vomeronasal 1 receptor 210	Pheromone binding	—	Inf	4.81
Sall1	sal-like 1 (Drosophila)	Transcriptional repressor	—	4.72	Inf
Prl2c3	prolactin family 2, subfamily c, member 3	Growth factor	—	5.03	7.05
Chil4	Chitinase-like 4	Chitin degradation	—	—	7.91
***Epidermal formation***					
Sprr2a1	small proline-rich protein 2A1	Keratinocyte envelope protein	7.64	5.85	5.99
Krt6b	Keratin 6b	Hair follicle formation	6.94	7.87	6.50
Spr2a3	small proline-rich protein 2A3	Keratinocyte envelope protein	6.58	5.14	6.29
Sprr2j-ps	small proline-rich protein 2J, pseudogene	Epithelial cell envelope	5.51	7.47	6.20
Sprr3	Small proline-rich protein 3	Keratinocyte envelope protein	—	Inf	5.98
Sprr2k	small proline-rich protein 2K	Epithelial cell envelope	—	8.00	7.97
***Unknown Function***					
Gm5581	Predicted gene 5581	Unknown	Inf	Inf	Inf
Gm14039	predicted gene 14039	Unknown	Inf	Inf	Inf
Gm24801	predicted gene, 24801	Unknown	Inf	Inf	Inf
Gm11345	predicted gene 11345	Unknown	Inf	Inf	Inf
Gm25465	predicted gene, 25465	Unknown	Inf	5.65	Inf
Gm15133	predicted gene 15133	Unknown	Inf	Inf	5.40
Fam71a	family with sequence similarity 71, member A	Unknown	Inf	Inf	4.83
Gm590	predicted gene 590	Unknown	4.69	Inf	Inf
Gm1966	predicted gene 1966	Unknown	Inf	6.18	3.28
Gm15845	predicted gene 15845	Unknown	Inf	5.07	6.46
Gm11956	Predicted gene 11956	Unknown	Inf	4.73	5.12
Tmem211	Transmembrane protein 211	Unknown	4.00	4.47	Inf
A530040E14RIK	Putative uncharacterized protein	Unknown	Inf	4.16	4.16
Gm16026	Predicted gene 16026	Unknown	Inf	3.10	4.48
Gm4847	Predicted gene 4847	Unknown	Inf	3.52	3.29
Gm5483	Predicted gene 5843	Unknown	7.84	6.90	6.25
2610528A11Rik	RIKEN cDNA 2610528A11 gene	Unknown	7.42	7.30	6.42
BC100530	cDNA sequence BC100530	Unknown	6.83	6.70	6.85
Gm5478	Predicted gene 5468	Unknown	6.54	7.24	5.97
BC117090	cDNA sequence BC1179090	Unknown	6.08	6.41	6.73
Gm5416	Predicted gene 5416	Unknown	5.97	7.70	6.08
1700012B09Rik	RIKEN cDNA 1700012B09 gene	Unknown	5.69	6.31	4.70
Gsdmc2	Gasdermin c2	Unknown	5.34	5.53	7.09
Gm15056	predicted gene 15056	Unknown	5.09	5.52	6.89
AC125149.1		Unknown	2.33	2.77	7.97
Gm20625	predicted gene 20625	Unknown	Inf	Inf	—
Gm9458	predicted gene 9458	Unknown	Inf	Inf	—
2010005H15Rik	RIKEN cDNA 2010005H15 gene	Unknown	Inf	4.85	—
Gm15729	Predicted gene 15729	Unknown	Inf	4.82	—
Gm10634	Predicted gene 10634	Unknown	Inf	4.33	—
4930519G04Rik	RIKEN cDNA 4930519G04 gene	Unknown	4.13	Inf	—
AC168977.1		Unknown	—	4.48	Inf
Psg18	pregnancy specific glycoprotein 18	Unknown	—	4.43	Inf
Gm13571	Predicted gene 13571	Unknown	—	Inf	4.17
Gm12253	Predicted gene 12253	Unknown	—	Inf	4.13
Gm11216	Predicted gene 11216	Unknown	—	Inf	3.51
Gm26686	predicted gene, 26686	Unknown	—	Inf	2.94
Gm14461	Predicted gnee 14461	Unknown	—	Inf	2.94
Gm11698	predicted gene 11698	Unknown	4.89	—	—

^1^Top 50 genes with greatest increase of LFC comparing infected ears to uninfected ears from challenged mice for each time point represented.

^2^Function determined via Entrez (www.ncbi.nlm.nih.gov) or Uniprot (www.uniprot.org).

^3^LFC = Log Fold Change.

**Table 2:** Top Genes with Significantly Increased Transcript Levels Systemically During SSTI^1^.

Symbol	Gene Name	Function^2^	LFC^3^		
			Day 1	Day 4	Day 7
***Immune Function***					
Cxcl2	chemokine (C-X-C motif) ligand 2	Chemokine	2.54	3.31	1.74
Cxcl3	chemokine (C-X-C motif) ligand 3	Chemokine	2.29	3.09	2.29
Irg1	immunoresponsive gene 1	Suppression of inflammation	2.17	3.35	1.95
S100a9	S100 calcium binding protein A9 (calgranulin B)	Component of calprotectin; regulation of immune response	1.19	1.77	—
IL-1b	interleukin 1 beta	Pro-inflammatory cytokine	—	1.65	1.37
Saa3	serum amyloid A 3	Acute phase protein	2.38	—	—
Crnn	cornulin	May play role in epithelial immune response	2.29	—	—
Cd8b1	CD8 antigen, beta chain 1	Coreceptor on cytotoxic T cells	2.10	—	—
Trem1	triggering receptor expressed on myeloid cells 1	Inflammation	—	2.86	—
Ccl20	chemokine (C-C motif) ligand 20	Chemotactic for lymphocytes	—	—	3.23
IL-20	interleukin 20	Cytokine	—	—	3.05
Retnlg	resistin like gamma	Potentially inflammatory hormone	—	—	2.10
Cxcl1	chemokine (C-X-C motif) ligand 1	Chemokine	—	—	2.05
Clec4e	C-type lectin domain family 4, member e	Pathogen receptor	—	—	1.96
Ccl4	chemokine (C-C motif) ligand 4	Chemokine	—	—	1.83
Chil1	chitinase-like 1	Potentially inflammatory	—	—	1.48
Tnf	tumor necrosis factor	Pro-inflammatory cytokine	—	—	1.43
Slfn4	schlafen 4	Immune cell development	—	—	1.38
Fos	FBJ osteosarcoma oncogene	Cellular development/inflammation in skin	—	—	1.15
Defb6	defensin beta 6	Antimicrobial	—	—	1.05
***Metabolism/Cell Proliferation/Regulation***					
mt-Co2	mitochondrially encoded cytochrome c oxidase II	Respiratory chain	—	2.75	1.13
mt-Atp6	mitochondrially encoded ATP synthase 6	ATP synthesis	—	2.74	1.12
mt-Co3	mitochondrially encoded cytochrome c oxidase III	Respiratory chain	—	2.74	1.12
Csn3	casein kappa	Stabilizes micelles	Inf	—	—
Kcne1	potassium voltage-gated channel, Isk-related subfamily, member 1	Potassium transport	3.11	—	—
Sct	secretin	Hormone	2.96	—	—
Slc5a5	solute carrier family 5 (sodium iodide symporter), member 5	Iodine uptake	2.30	—	—
Slc4a1	solute carrier family 4 (anion exchanger), member 1	Anion exchange in erythrocytes	2.21	—	—
Crisp1	cysteine-rich secretory protein 1	Sperm-egg fusion	2.17	—	—
Kcnk16	potassium channel, subfamily K, member 16	Outward rectifying potassium channel	2.14	—	—
Cldn14	claudin 14	Tight junction protein	2.11	—	—
mt-Tc	mitochondrially encoded tRNA cysteine	RNA gene	—	5.14	—
mt-Nd4l	mitochondrially encoded NADH dehydrogenase 4L	Electron transport	—	4.72	—
mt-Atp8	mitochondrially encoded ATP synthase 8	Electron transport	—	4.36	—
Aldoart2	aldolase 1 A, retrogene 2	Glyocolysis	—	3.84	—
Pon1	paraoxonase 1	Enzyme	—	2.45	—
Galnt2	UDP-N-acetyl-alpha-D-galactosamine:polypeptide N-acetylgalactosaminyltransferase 2	Protein glycosylation	—	2.21	—
mt-Nd3	mitochondrially encoded NADH dehydrogenase 3	Respiratory chain	—	2.13	—
Adrb3	adrenergic receptor, beta 3	Regulation of lipolysis	—	2.11	—
Thrsp	thyroid hormone responsive	Regulation of lipogenesis	—	1.83	—
Chat	choline acetyltransferase	Acetylcholine production	—	1.59	—
Gsdma3	gasdermin A3	Pro-apoptotic	—	—	1.35
Syt4	synaptotagmin IV	Cellular transport	—	—	1.01
Gpr128	G protein-coupled receptor 128	Receptor	*-3*.*42*	2.37	—
Rpph1	ribonuclease P RNA component H1	Endonuclease	—	3.83	*-3*.*25*
Rnu3a	U3A small nuclear RNA	Non-coding RNA	—	3.10	*-2*.*43*
Snord17	small nucleolar RNA, C/D box 17	Non-coding RNA	—	3.07	*-2*.*60*
Rn7sk	RNA, 7SK, nuclear	Pre-mRNA processing	—	3.02	*-2*.*59*
Yam1	YY1 associated myogenesis RNA 1	Non-coding RNA	—	1.95	*-1*.*57*
Gys2	glycogen synthase 2	Glycogen production	—	1.85	*-1*.*86*
Lars2	leucyl-tRNA synthetase, mitochondrial	tRNA synthesis	—	1.83	*-1*.*41*
***Epidermal Formation***					
Krtap20-2	keratin associated protein 20–2	Hair formation	3.34	—	—
Krt72	keratin 72	Hair formation	3.02	—	—
Krtap9-5	keratin associated protein 9–5	Hair formation	2.83	—	—
Krtap31-2	keratin associated protein 31–2	Hair formation	2.72	—	—
Krt26	keratin 26	Hair formation	2.59	—	—
Krtap4-2	keratin associated protein 4–2	Hair formation	2.56	—	—
Krt28	keratin 28	Hair formation	2.47	—	—
Krtap4-13	keratin associated protein 4–13	Hair formation	2.47	—	—
Krtap31-1	keratin associated protein 31–1	Hair formation	2.40	—	—
Krt74	keratin 74	Hair formation	2.33	—	—
Tchhl1	trichohyalin-like 1	Hair formation	2.23	—	—
Krtap4-9	keratin associated protein 4–9	Hair formation	2.18	—	—
Krt27	keratin 27	Epithelial cytoskeleton	2.17	—	—
Tchh	trichohyalin	Epithelial tissue strength	2.14	—	—
Padi1	peptidyl arginine deiminase, type I	Epidermal differentiation	2.02	—	—
Fgf5	fibroblast growth factor 5	Regulation of hair growth	2.01	—	—
Padi3	peptidyl arginine deiminase, type III	Modulates hair structural proteins	1.99	—	—
Krtap4-8	keratin associated protein 4–8	Hair formation	1.95	—	—
Stfa2l1	stefin A2 like 1	Epidermal development	—	2.35	—
Krtap19-1	keratin associated protein 19–1	Hair formation	—	1.60	—
***Unknown Function***					
Gm10243	predicted gene 10243	Unknown	—	2.85	1.64
Rps11-ps4	ribosomal protein S11, pseudogene 4	Pseudogene	—	2.27	1.50
Rpl19-ps1	ribosomal protein L19, pseudogene 1	Pseudogene	—	1.85	1.37
Gm8810	predicted gene 8810	Unknown	—	1.77	1.65
Gm9789	predicted gene 9789	Unknown	4.26	—	—
Gm11569	predicted gene 11569	Unknown	4.25	—	—
Gm14180	predicted gene 14180	Unknown	3.64	—	—
Gm11564	predicted gene 11564	Unknown	3.62	—	—
Gm11554	predicted gene 11554	Unknown	3.61	—	—
D130052B06Rik	RIKEN cDNA D130052B06 gene	Unknown	3.37	—	—
Gm14182	predicted gene 14182	Unknown	3.23	—	—
1110057P08Rik	RIKEN cDNA 1110057P08 gene	Unknown	3.07	—	—
Gm6358	predicted gene 6358	Unknown	2.98	—	—
Gm11596	predicted gene 11596	Unknown	2.87	—	—
Gm7735	predicted gene 7735	Unknown	2.69	—	—
Gm5278	predicted pseudogene 5278	Unknown	2.67	—	—
Gm10061	predicted gene 10061	Unknown	2.48	—	—
2300002M23Rik	RIKEN cDNA 2300002M23 gene	Unknown	2.21	—	—
Gm11595	predicted gene 11595	Unknown	2.08	—	—
Crym	crystallin, mu	Unknown	2.04	—	—
Gm11563	predicted gene 11563	Unknown	1.93	—	—
Gm14513	predicted gene 14513	Unknown	—	4.30	—
Mup-ps22	major urinary protein, pseudogene 22	Pseudogene	—	3.56	—
Rps13-ps1	ribosomal protein S13, pseudogene 1	Pseudogene	—	3.46	—
Gm5483	predicted gene 5483	Unknown	—	2.79	—
Gm12883	predicted gene 12883	Unknown	—	2.69	—
Mup18	major urinary protein 18	Unknown	—	2.40	—
Gm14323	predicted gene 14323	Unknown	—	—	2.02
Rpl31-ps16	ribosomal protein L31, pseudogene 16	Pseudogene	—	—	1.84
Rps15a-ps3	ribosomal protein S15A, pseudogene 3	Pseudogene	—	—	1.84
Gm10132	predicted gene 10132	Unknown	—	—	1.83
Slpi	secretory leukocyte peptidase inhibitor	Unknown	—	—	1.82
Gm13675	predicted gene 13675	Unknown	—	—	1.70
Gm5453	predicted gene 5453	Unknown	—	—	1.70
Calr-ps	calreticulin, pseudogene	Pseudogene	—	—	1.34
Rpl31-ps11	ribosomal protein L31, pseudogene 11	Pseudogene	—	—	1.33
Gm8080	predicted gene 8080	Unknown	—	—	1.20
Gm12482	predicted gene 12482	Unknown	—	—	1.14
Rpl13-ps3	ribosomal protein L13, pseudogene 3 [	Pseudogene	—	—	1.02
Gm12248	predicted gene 12248	Unknown	*-1*.*06*	—	1.21
Gm24407	predicted gene, 24407	Unknown	2.19	4.66	*-4*.*01*
Gm24265	predicted gene, 24265	Unknown	1.74	4.93	*-3*.*54*
Rprl3	ribonuclease P RNA-like 3	Unknown	—	Inf	*-3*.*14*
Gm14279	predicted gene 14279	Unknown	*-1*.*65*	5.34	—
Gm24270	predicted gene, 24270	Unknown	—	2.77	*-1*.*88*
Gm24187	predicted gene, 24187	Unknown	—	2.49	*-2*.*47*
Gm23935	predicted gene, 23935	Unknown	—	3.55	*-2*.*31*
Gm15564	predicted gene 15564	Unknown	—	2.21	*-1*.*31*
BC100530	cDNA sequence BC100530	Unknown	*-1*.*74*	1.62	*-1*.*18*

^1^Top 50 genes with greatest increase of LFC comparing uninfected ears from challenged mice to naïve mice for each time point represented; for Day 7, all genes with significant levels of transcript increases are listed.

^2^Function determined via Entrez (www.ncbi.nlm.nih.gov) or Uniprot (www.uniprot.org)

^3^LFC = Log Fold Change

Italicized values indicate transcripts are significantly decreased at the indicated time point.

**Table 3:** Top Genes with Significantly Decreased Transcript Levels at the Site of SSTI^1^.

Symbol	Gene Name	Function^2^	LFC^3^		
			Day 1	Day 4	Day 7
***Immune Function***					
Fcer2a	Fc receptor, IgE, low affinity II, alpha polypeptide	Receptor for IgE; B cell differentiation	-1.61	-1.87	-1.50
Serpinb1c	serine (or cysteine) peptidase inhibitor, clade B, member 1c	Inhibits neutrophil-derived proteinases	—	-1.11	-1.65
Marco	macrophage receptor with collagenous structure	Pattern recognition receptor	—	-1.61	—
Bpifb2	BPI fold containing family B, member 2	LPS binding	—	—	-2.10
Lyg2	lysozyme G-like 2	cleaves peptidoglycan	—	—	-1.67
***Metabolism/Cell Proliferation/Regulation***					
Inmt	indolethylamine N-methyltransferase	Indole N-methylation	-4.40	-1.63	-1.33
Nell2	NEL-like 2	Protein kinase C binding	-1.81	-1.81	-1.60
Cyp11b1	cytochrome P450, family 11, subfamily b, polypeptide 1	Conversion of progesterone to cortisol	-1.45	-1.88	-2.04
Sost	sclerostin	Negative regulator of bone growth	-3.43	-2.06	—
Mrgprg	MAS-related GPR, member G	Pain sensation/modulation	-3.22	-1.65	—
Gkn3	gastrokine 3	May inhibit gastric cell proliferation	-3.05	-1.63	—
Spock3	sparc/osteonectin, cwcv and kazal-like domains proteoglycan 3	Inhibition of matrix metalloproteinase processing	-2.96	-1.82	—
Hrk	harakiri, BCL2 interacting protein (contains only BH3 domain)	Pro-apoptotic	-2.64	-2.19	—
Thrsp	thyroid hormone responsive	Regulation of lipogenesis	-2.35	-1.18	—
Mettl11b	methyltransferase like 11B	Methyltransferase	-2.25	-1.95	—
Cntnap2	contactin associated protein-like 2	Neurogenesis	-2.23	-1.59	—
Gucy2f	guanylate cyclase 2f [Source:MGI Symbol;Acc:MGI:105119]	cGMP resynthesis	-1.49	-1.87	—
Ms4a10	membrane-spanning 4-domains, subfamily A, member 10	Signal transduction	-1.35	-1.61	—
Sun5	Sad1 and UNC84 domain containing 5	Spermatogenesis	—	-4.05	-2.29
Myl3	myosin, light polypeptide 3	Light chain of myosin	—	-2.03	-1.45
Ptgds	prostaglandin D2 synthase (brain)	Prostaglandin synthesis	—	-1.97	-1.22
Oxct2b	3-oxoacid CoA transferase 2B	Metabolism	-3.91	—	—
Gmnc	geminin coiled-coil domain containing	Regulation of DNA replication	-3.35	—	—
Odf4	outer dense fiber of sperm tails 4	Spermatogenesis	-3.27	—	—
Cntn4	contactin 4	Neuronal development	-2.72	—	—
Rergl	RERG/RAS-like	GTPase	-2.67	—	—
Mettl21e	methyltransferase like 21E	Lysine methyltransferase	-2.65	—	—
Prss51	protease, serine 51	Endopeptidase	-2.57	—	—
Mylk4	myosin light chain kinase family, member 4	Muscle development	-2.55	—	—
Unc5d	unc-5 homolog D (C. elegans)	Netrin receptor; may be required for apoptosis	-2.52	—	—
Mrgprh	MAS-related GPR, member H	G protein coupled receptor	-2.47	—	—
Olfr1420	olfactory receptor 1420	Sensory perception of smell	-2.44	—	—
Abca6	ATP-binding cassette, sub-family A (ABC1), member 6	Probable transporter	-2.40	—	—
Fhl5	four and a half LIM domains 5	Transcriptional activator	-2.30	—	—
Nlgn1	neuroligin 1	Synapse formation	-2.26	—	—
Ocm	oncomodulin	Calcium binding regulator	-2.24	—	—
Kcnt1	potassium channel, subfamily T, member 1	Potassium transport	-2.21	—	—
Ky	kyphoscoliosis peptidase	Muscle growth	-2.21	—	—
mt-Atp8	mitochondrially encoded ATP synthase 8	Electron transport	—	-5.32	—
mt-Co3	mitochondrially encoded cytochrome c oxidase III	Electron transport	—	-3.73	—
mt-Co2	mitochondrially encoded cytochrome c oxidase II	Electron transport	—	-3.49	—
Aldoart2	aldolase 1 A, retrogene 2	Glyocolysis	—	-3.32	—
mt-Tc	mitochondrially encoded tRNA cysteine	RNA gene	—	-3.06	—
mt-Atp6	mitochondrially encoded ATP synthase 6	ATP synthesis	—	-3.02	—
mt-Nd3	mitochondrially encoded NADH dehydrogenase 3	Respiratory chain	—	-2.76	—
Dbx1	developing brain homeobox 1	Neurogenesis	—	-2.74	—
Efcab6	EF-hand calcium binding domain 6	Negatively regulates androgen receptor	—	-1.82	—
Myl2	myosin, light polypeptide 2, regulatory, cardiac, slow	Muscle development	—	-1.64	—
Kif12	kinesin family member 12	Intracellular transport	—	—	-2.17
Lrp1b	low density lipoprotein-related protein 1B (deleted in tumors)	Cell surface protein that may bind ligands for endocytosis	—	—	-2.01
Actbl2	actin, beta-like 2	Cell motility	—	—	-1.94
***Epidermal Formation***					
Ros1	Ros1 proto-oncogene	Epithelial cell differentiation	-2.19	-2.26	-2.04
Krt24	keratin 24	Epithelial cytoskeleton	—	-1.68	-1.66
Krtap11-1	keratin associated protein 11–1	Hair formation	—	-1.05	-1.65
Krtap26-1	keratin associated protein 26–1	Hair formation	—	-1.05	-1.60
Krtap4-8	keratin associated protein 4–8	Hair formation	—	-1.03	-1.65
Krtap4-13	keratin associated protein 4–13	Hair formation	—	—	-2.04
Krtap12-1	keratin associated protein 12–1	Hair formation	—	—	-1.95
Krtap16-1	keratin associated protein 16–1	Hair formation	—	—	-1.87
Krtap24-1	keratin associated protein 24–1	Hair formation	—	—	-1.75
Krt82	keratin 82	Hair formation	—	—	-1.72
Krtap10-4	keratin associated protein 10–4	Hair formation	—	—	-1.69
Krtap4-9	keratin associated protein 4–9	Hair formation	—	—	-1.69
Krtap10-10	keratin associated protein 10–10	Hair formation	—	—	-1.65
Krtap1-3	keratin associated protein 1–3	Hair formation	—	—	-1.62
Krtap1-4	keratin associated protein 1–4	Hair formation	—	—	-1.62
Krtap5-5	keratin associated protein 5–5	Hair formation	—	—	-1.60
***Unknown Function***					
4921504E06Rik	RIKEN cDNA 4921504E06 gene	Unknown	-2.27	-2.25	-1.98
1190003K10Rik	RIKEN cDNA 1190003K10 gene	Unknown	-1.98	-1.74	-1.50
Gm4810	predicted gene 4810	Unknown	-1.90	-2.53	-2.30
5430427M07Rik	RIKEN cDNA 5430427M07 gene	Unknown	-1.74	-1.79	-1.23
Fam150b	family with sequence similarity 150, member B	Unknown	-2.19	-1.60	—
Serpina4-ps1	serine (or cysteine) peptidase inhibitor, clade A, member 4, pseudogene 1	Pseudogene	-1.67	-1.70	—
Smim18	small integral membrane protein 18	Unknown	-1.57	-2.11	—
Fam196b	family with sequence similarity 196, member B	Unknown	-1.40	-1.69	—
2410137M14Rik	RIKEN cDNA 2410137M14 gene	Unknown	-1.29	-1.63	—
Gm11433	predicted gene 11433	Unknown	—	-1.80	-1.91
Gm8810	predicted gene 8810	Unknown	—	-1.67	1.00
Gm11564	predicted gene 11564	Unknown	—	-1.55	-2.39
2310005G13Rik	RIKEN cDNA 2310005G13 gene	Unknown	—	-1.53	-1.59
Gm11568	predicted gene 11568	Unknown	—	-1.03	-1.64
1700019B03Rik	RIKEN cDNA 1700019B03 gene	Unknown	-1.27	—	-1.68
Arrdc5	arrestin domain containing 5	Unknown	-3.95	—	—
Gm13441	predicted gene 13441	Unknown	-3.56	—	—
Gm17025	predicted gene 17025	Unknown	-3.47	—	—
A330094K24Rik	RIKEN cDNA A330094K24 gene	Unknown	-3.25	—	—
Tsga13	testis specific gene A13	Unknown	-3.01	—	—
Gm13273	predicted gene 13273	Unknown	-2.85	—	—
Gm1305	predicted gene 1305	Unknown	-2.83	—	—
Gm14411	predicted gene 14411	Unknown	-2.78	—	—
Teddm1	transmembrane epididymal protein 1	Unknown	-2.60	—	—
Gm27195	predicted gene 27195	Unknown	-2.59	—	—
A230108P19Rik	RIKEN cDNA A230108P19 gene	Unknown	-2.56	—	—
Gm9947	predicted gene 9947	Unknown	-2.56	—	—
1700018A04Rik	RIKEN cDNA 1700018A04 gene	Unknown	-2.47	—	—
Prr32	proline rich 32	Unknown	-2.39	—	—
BB218582	expressed sequence BB218582	Unknown	-2.39	—	—
1700119I11Rik	RIKEN cDNA 1700119I11 gene	Unknown	-2.38	—	—
1600029O15Rik	RIKEN cDNA 1600029O15 gene	Unknown	-2.34	—	—
Lrrc30	leucine rich repeat containing 30	Unknown	-2.32	—	—
Tcerg1l	transcription elongation regulator 1-like	Unknown	-2.23	—	—
Gm14513	predicted gene 14513	Unknown	—	-5.22	—
Gm5590	predicted gene 5590	Unknown	—	-4.83	—
Mup-ps22	major urinary protein, pseudogene 22	Pseudogene	—	-3.13	—
Rps13-ps1	ribosomal protein S13, pseudogene 1	Pseudogene	—	-2.86	—
Rpl31-ps16	ribosomal protein L31, pseudogene 16	Pseudogene	—	-2.49	—
Rps11-ps4	ribosomal protein S11, pseudogene 4	Pseudogene	—	-2.42	—
Rpl19-ps1	ribosomal protein L19, pseudogene 1	Pseudogene	—	-2.01	—
Gm17597	predicted gene, 17597	Unknown	—	-1.72	—
Gm14279	predicted gene 14279	Unknown	*2*.*08*	-1.86	*4*.*76*
Gm11571	predicted gene 11571	Unknown	—	—	-2.73
G630018N14Rik	RIKEN cDNA G630018N14 gene	Unknown	—	—	-2.03
Gm9507	predicted gene 9507	Unknown	—	—	-1.94
Fam26d	family with sequence similarity 26, member D	Unknown	—	—	-1.93
Gm3250	predicted gene 3250	Unknown	—	—	-1.93
Gm7579	predicted gene 7579	Unknown	—	—	-1.84
Gm10100	predicted gene 10100	Unknown	—	—	-1.81
Gm11555	predicted gene 11555	Unknown	—	—	-1.79
Gm3233	predicted gene 3233	Unknown	—	—	-1.78
Gm7138	predicted gene 7138	Unknown	—	—	-1.77
Gm19402	predicted gene, 19402	Unknown	—	—	-1.75
Gm11567	predicted gene 11567	Unknown	—	—	-1.74
Gm2431	predicted gene 2431	Unknown	—	—	-1.73
Gm4559	predicted gene 4559	Unknown	—	—	-1.69
Gm3238	predicted gene 3238	Unknown	—	—	-1.62
Gm11596	predicted gene 11596	Unknown	—	—	-1.61
Gm10318	predicted gene 10318	Unknown	—	—	-1.56
Cdh7	cadherin 7, type 2	Adhesion	-Inf	—	—
Dlx6os2	distal-less homeobox 6, opposite strand 2	Unknown	-Inf	—	—
mt-Nd4l	mitochondrially encoded NADH dehydrogenase 4L		—	-Inf	—

^1^Top 50 genes with greatest negative change in LFC when comparing infected ears to uninfected ears from challenged mice for each time point represented

^2^Function determined via Entrez (www.ncbi.nlm.nih.gov) or Uniprot (www.uniprot.org)

^3^LFC = Log Fold Change

Italicized values indicate transcripts are significantly increased at the indicated time point.

**Table 4:** Top Genes with Significantly Decreased Transcript Levels Systemically During SSTI^1^.

Symbol	Gene Name	Function^2^	LFC^3^		
			Day 1	Day 4	Day 7
***Immune Function***					
Serpinb3a	serine (or cysteine) peptidase inhibitor, clade B (ovalbumin), member 3A	May modulate immune response and apoptosis	-2.80	*1*.*02*	—
Spon2	spondin 2, extracellular matrix protein	Adhesin; can function as opsonin for phagocytosis of bacteria	-1.04	—	—
Glycam1	glycosylation dependent cell adhesion molecule 1	Ligand for L-selectin	—	—	-4.55
Ighg2c	immunoglobulin heavy constant gamma 2C	Immunoglobulin	—	—	-3.62
Reg3g	regenerating islet-derived 3 gamma	Anti-bacterial C-type lectin	—	—	-3.27
Pax5	paired box 5	B cell differentiation	—	—	-3.16
Igkc	immunoglobulin kappa constant	Immunoglobulin	—	—	-2.81
Chil4	chitinase-like 4	Chemotactic for eosinophils; potentially inflammatory	—	—	-2.97
Ms4a1	membrane-spanning 4-domains, subfamily A, member 1	Regulation of B cell activation/proliferation	—	—	-2.34
Hamp2	hepcidin antimicrobial peptide 2	Antimicrobial; iron storage regulation	—	—	-2.18
Cd22	CD22 antigen	Mediates B-B cell interactions	—	—	-1.94
Cd99	CD99 antigen	T cell adhesion	—	—	-1.19
***Metabolism/Cell Proliferation/Regulation***					
Gpr128	G protein-coupled receptor 128	Receptor	-3.42	*2*.*37*	—
Serpina3k	serine (or cysteine) peptidase inhibitor, clade A, member 3K	Protease inhibitor	-3.24	—	—
Jakmip2	janus kinase and microtubule interacting protein 2	Structural scaffold of Golgi	-2.48	—	—
Otor	otoraplin	Cartilage development	-2.19	—	—
Spink8	serine peptidase inhibitor, Kazal type 8	Serine protease inhibitor	-1.93	—	—
Akr1c19	aldo-keto reductase family 1, member C19	Metabolism	-1.89	—	—
Mmp13	matrix metallopeptidase 13	Extracellular matrix degradation	-1.65	—	—
Epyc	epiphycan	Bone/cartilage formation	-1.20	—	—
Sfrp4	secreted frizzled-related protein 4	Regulation of cell growth	-1.09	—	—
Cyp26a1	cytochrome P450, family 26, subfamily a, polypeptide 1	Retinoic acid metabolism	-1.03	—	—
Akr1c18	aldo-keto reductase family 1, member C18	Metabolism	-1.02	—	—
Cyp2a5	cytochrome P450, family 2, subfamily a, polypeptide 5	Metabolism	—	-1.58	—
Nr1h4	nuclear receptor subfamily 1, group H, member 4	Transcription factor	*1*.*49*	-1.46	—
Serpina3j	serine (or cysteine) peptidase inhibitor, clade A, member 3J	Protease inhibitor	—	-1.05	—
Rpph1	ribonuclease P RNA component H1	tRNA formation	—	*3*.*83*	-3.25
Gdf7	growth differentiation factor 7	Neuronal development	—	—	-2.94
Reg1	regenerating islet-derived 1	Metabolism	—	—	-2.85
Snord17	small nucleolar RNA, C/D box 17	Non-coding RNA	—	*3*.*07*	-2.60
Rn7sk	RNA, 7SK, nuclear	Non-coding RNA	—	*3*.*06*	-2.59
Bsnd	Bartter syndrome, infantile, with sensorineural deafness (Barttin)	Chloride channel formation	—	—	-2.45
Rnu3a	U3A small nuclear RNA	Non-coding RNA	—	*3*.*10*	-2.43
Pyy	peptide YY	Reduces pancreatic secretions; vasoconstrictory	—	—	-2.39
Serpinb6e	serine (or cysteine) peptidase inhibitor, clade B, member 6e	Inner ear; protects against lysosomal leakage during stress	—	—	-1.96
Gys2	glycogen synthase 2	Glycogen synthesis	—	*1*.*85*	-1.86
Yam1	YY1 associated myogenesis RNA 1	Non-coding RNA	—	*1*.*95*	-1.57
Ntrk1	neurotrophic tyrosine kinase, receptor, type 1	Nervous system development	—	—	-1.48
Lars2	leucyl-tRNA synthetase, mitochondrial	tRNA formation	—	*1*.*83*	-1.41
Hist1h2al	histone cluster 1, H2al	Nucleosome component	—	—	-1.38
Scand1	SCAN domain-containing 1	Transcriptional regulator	—	—	-1.38
***Epidermal Development***					
H60c	histocompatibility 60c	Epithelial integrity	-1.01	—	—
Stfa2	stefin A2	Cysteine protease inhibitor/Epidermal development	—	—	-1.65
Krt6b	keratin 6B	Epithelial development	—	—	-1.50
Sprr1b	small proline-rich protein 1B	Keratinocyte protein	—	—	-1.07
***Unknown***					
Cyp2g1	cytochrome P450, family 2, subfamily g, polypeptide 1	Pseudogene	—	-2.81	-2.31
Gm8221	predicted gene 8221	Unknown	—	-2.50	-2.20
Sprr2a2	small proline-rich protein 2A2	Unknown	-2.06	—	-3.41
Gsdmc	gasdermin C	Unknown	-1.89	—	-2.03
Sprr2a3	small proline-rich protein 2A3	Unknown	-1.86	—	-2.10
BC100530	cDNA sequence BC100530	Unknown	-1.74	*1*.*62*	-1.20
Mup9	major urinary protein 9	Unknown	—	*1*.*05*	-1.36
Gm5478	predicted pseudogene 5478	Unknown	—	—	-1.34
Gm6484	predicted gene 6484	Unknown	—	—	-1.34
Gm15564	predicted gene 15564	Unknown	—	*2*.*21*	-1.31
TMEM134	transmembrane protein 134 (Tmem134), transcript variant 1, mRNA	Unknown	—	—	-1.30
2610528A11Rik	RIKEN cDNA 2610528A11 gene	Unknown	—	—	-1.27
Rps2-ps10	ribosomal protein S2, pseudogene 10	Pseudogene	—	—	-1.21
Apol11b	apolipoprotein L 11b	Unknown	-3.04	—	—
Gm14279	predicted gene 14279	Unknown	-1.65	*5*.*34*	—
Gm12248	predicted gene 12248	Unknown	-1.07	—	*1*.*21*
Rps8-ps2	ribosomal protein S8, pseudogene 2	Pseudogene	—	-3.71	—
Rpl15-ps2	ribosomal protein L15, pseudogene 2	Pseudogene	—	-2.08	—
Gm9581	predicted gene 9581	Unknown	—	-1.38	—
Apol9a	apolipoprotein L 9a	Unknown	—	-1.25	—
Gm24407	predicted gene, 24407	Unknown	*2*.*19*	*4*.*66*	-4.01
Gsdmc2	gasdermin C2	Unknown	—	—	-3.89
Gm25360	predicted gene, 25360	Unknown	—	—	-3.81
Gm24265	predicted gene, 24265	Unknown	*1*.*74*	*4*.*93*	-3.54
Rprl3	ribonuclease P RNA-like 3	Unknown	—	*Inf*	-3.14
Gm19980	predicted gene, 19980	Unknown	—	—	-2.49
Gm24187	predicted gene, 24187	Unknown	—	*2*.*49*	-2.47
Akr1cl	aldo-keto reductase family 1, member C-like	Unknown	—	—	-2.43
Mup7	major urinary protein 7	Unknown	—	—	-2.36
Gm23935	predicted gene, 23935	Unknown	—	*3*.*54*	-2.31
Gm4841	predicted gene 4841	Unknown	—	—	-1.95
BC117090	cDNA sequence BC1179090	Unknown	—	—	-1.95
Gm24270	predicted gene, 24270	Unknown	—	*2*.*77*	-1.88
Gm26917	predicted gene, 26917	Unknown	—	—	-1.58
Gm21887	predicted gene, 21887	Unknown	—	—	-1.45

^1^All genes that had a significantly decreased LFC when comparing uninfected ears from challenged mice to naïve mice are listed.

^2^Function determined via Entrez (www.ncbi.nlm.nih.gov) or Uniprot (www.uniprot.org)

^3^LFC = Log Fold Change

Italicized values indicate transcripts are significantly increased at the indicated time point.

### Upstream Regulator Analysis

In order to identify which signaling pathways were potentially activated or repressed during *S*. *aureus* SSTI, we used the Upstream Regulator Analytic (URA) of IPA (Ingenuity Systems, www.ingenuity.com). This software evaluates the overlap among experimentally derived gene lists and the Ingenuity Knowledge Base, a comprehensive database of genetic data containing approximately 5 million findings manually selected from the scientific literature or third party databases [18]. URA calculates the statistical significance of the overlap between the experimental dataset and the database’s regulatory pathways and reports a *p* value using Fisher’s Exact Test. URA also analyzes the direction of the differential gene expression to predict the activation or repression of these pathways (for example, if Regulator X is reported in the literature to cause up-regulation of genes A, B, C, and D, and these four genes are found experimentally to have significantly increased transcript levels, URA will predict the pathway regulated by X is activated; if genes A, B, C, and D have significantly decreased transcript levels, URA will predict the pathway is inhibited) and reports an Activation Z-score. Activation Z-scores ≥2 are considered “activated” and scores ≤2 are considered “inhibited”.

### Sequencing Data Access

All raw sequencing reads have been submitted to the NCBI Sequence Read Archive (http://www.ncbi.nlm.nih.gov/sra) under the accession number SRP040121. The processed gene expression data have been submitted to the NCBI Gene Expression Omnibus (http://www.ncbi.nlm.nih.gov/geo/) under accession number GSE56227.

### Western Blotting

Ears were harvested as indicated above and placed into 1 mL of lysis buffer (5 mM Tris pH 8, 150 mM NaCl, 1% NP-40 [Surfact-Amps, ThermoFisher], 1x protease inhibitor cocktail [Halt, ThermoFisher]) on ice, and then homogenized. Ear homogenates were centrifuged at 12000 × *g* for 10 minutes. The protein concentration of the supernatants was determined using BCA (Pierce BCA Protein Assay Kit, ThermoFisher), and lysates were kept at -80°C until use.

For Western blots, 15 μg total protein content per lane was resolved on 10–20% Tris-Glycine gels (Novex, ThermoFisher) or 4–12% Bis-Tris gels (NuPage, ThermoFisher). Proteins were transferred to nitrocellulose using the iBlot system and blots were blocked for 1 hour with 5% milk in TBS with 0.1% Tween 20 (TBS-T) or PBS with 0.1% Tween 20 (PBS-T) at RT with gentle shaking. Blots were then incubated with primary antibodies (anti-IL1β Mouse mAb #12242, Cell Signaling Technology, Danvers, MA; anti-S100A8/Calgranulin A pAb sc-8112, anti-S100A8/Calgranulin B pAB sc-8115, and anti-GAPDH pAb sc-20357, Santa Cruz Biotechnology, Dallas, TX) overnight at 4°C in TBS-T with 5% BSA (IL-1β, S100A9, and GAPDH), or for 1 hour at RT in PBS-T with 5% milk (S100A8). Blots were washed three times for 10 minutes each in TBS-T or PBS-T, and then incubated with HRP-conjugated goat anti-mouse IgG (IL-1β; KPL, Gaithersburg, MD) or donkey anti-goat IgG (S100A8, S100A9, and GAPDH; Santa Cruz Biotechnology) diluted in TBS-T or PBS-T for one hour at RT. Blots were imaged using ECL (GE Healthcare, Pittsburgh, PA).

## Results

### RNA-Seq of murine skin during *S*. *aureus* SSTI

We compared cDNA profiles of infected and uninfected murine ears at one, four, and seven days post-challenge. Sequencing reads were aligned to the mouse genome and were analyzed as described in the Materials and Methods. We defined differentially expressed genes as having a minimum of two-fold change in expression (log_2_ fold change ≥1, *P* < 0.01) when comparing infected ears to uninfected ears from challenged mice to evaluate the local response at the site of infection, or when comparing uninfected ears from challenged mice to naïve mice to examine the systemic response.

We found 1477, 1298, and 1351 genes with significantly increased transcript levels in infected ears compared to non-infected ears from the same challenged mice at days one, four, and seven, respectively. There were 585 genes that exhibited significantly decreased transcript levels at the site of infection (comparing infected ears compared to uninfected ears from challenged mice) at Day 1, 195 at Day 4, and 143 at Day 7. The complete list is available in S1 Table. In order to evaluate the systemic response, we also compared the RNA-seq profiles of the uninfected ears from challenged mice to those of naïve mice. In this analysis we found 123 genes with significantly increased transcript levels at Day 1, 66 at Day 4, and 38 at Day 7. We found 21 genes with significantly decreased transcript levels in uninfected ears from challenged mice compared to naïve mice at Day 1, 9 at Day 4, and 56 at Day 7 (S1 Table). These results demonstrate that the challenged mice exhibit a higher level of differential gene expression locally at the infection site than at more distal sites.

Because of the large amount of data generated in these studies, the top 50 differentially expressed genes at each tested time point are presented in Tables 1–4. These tables were compiled by sorting the gene expression data for each comparison (infected ears to non-infected ears from challenged mice for the local response, and non-infected ears from challenged mice to naïve mice for the systemic response) at each time point (Day 1, 4, and 7), and listing the 50 genes for each that had the highest LFC for genes with increased transcript levels, or the lowest LFC for genes that had decreased transcript levels. For each of the top 50 genes, we then determined the LFC at the other time points and added these values to the tables to show the gene’s change in expression over time. Many genes were highly altered in transcript levels at more than one time point; thus, none of Tables 1–4 contain 150 genes (50 for each of the time points).

**Fig 1:**
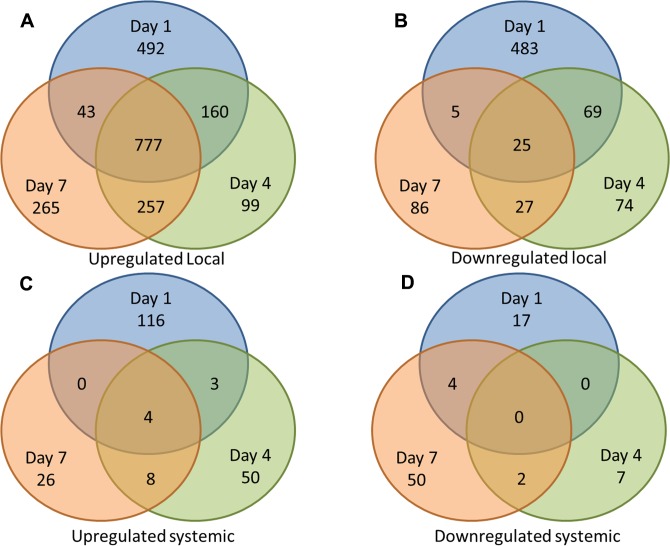
Comparative levels of differentially expressed genes over time in a murine staphylococcal SSTI model. Venn diagrams depicting the number of significantly changed (LFC ≥ 1 or ≤-1) transcripts at day 1 (blue), day 4 (green), and day 7 (orange) are shown. Transcripts that are common to multiple time points are shown by the overlap. (A) Significantly increased transcripts in infected ears compared to non-infected ears from the same challenged mice (local response); (B) Significantly decreased transcripts in infected ears compared to non-infected ears from the same challenged mice (local response); (C) Significantly increased transcripts in the non-infected ears of challenged mice compared to naïve mice (systemic response); (D) Significantly decreased transcripts in the non-infected ears of challenged mice compared to naïve mice (systemic response).

Table 1 contains the top genes with the highest increases in transcript levels for each time point during the local response, where we compared infected ears at each time point to the corresponding uninfected ears from the same mice. Of these top genes, 39 encode proteins that have known functions in the immune response, including 17 cytokines, chemokines, and chemokine-like proteins, as well as *Cxcr1*, which encodes an IL-8 receptor. Several genes encoding cytolytic granule proteins (*Olfm4*, *Mcpt1*, and *Gzmk*) were also top up-regulated immune genes at least at one tested time point during SSTI. Overall, the majority of genes with a known immune function that have the greatest increases at the site of infection appear to have a role in the innate response. Table 1 also shows that 22 genes encoding proteins involved in metabolism, cell proliferation, and regulation are among the genes with the greatest transcript increases at the site of infection. We also found six genes encoding epidermal proteins, and 39 uncharacterized genes, in the top 50 list of genes with the highest increases in transcript levels. We also note that the majority of the genes in Table 1 had significantly increased transcript levels at more than one tested time point, suggesting that the response is consistent over time. This is reflected in the overall list of genes with locally significant increases in transcript levels (S1 Table), with 60% of these genes demonstrating significant increases in transcript levels at multiple times (Fig 1A).

Table 2 contains the genes that demonstrated the greatest increases in transcript levels systemically at each tested time point; these genes were differentially expressed when comparing the uninfected ears from challenged mice to those of naïve mice. Of these genes, 20 encode proteins with an immune function. The immune genes that are common between the local and systemic responses are *Cxcl2*, *Cxcl3*, *Irg1*, *S100a9*, *Saa3*, *Il-1β*, *Trem1*, and *Clec4e*. For each of these genes, the LFC in the local response is higher than it is in the systemic response (i.e. 8.97 vs 2.29 for the LFC of *Cxcl3* at Day 1 locally and systemically, respectively). Also, while transcript levels for each of these common genes are consistently increased at each time point in the local response, only *Cxcl2*, *Cxcl3*, and *Irg1* are significantly increased at all three time points when looking at the systemic response. The higher number of top genes that encode proteins that function in the immune response, as well as their greater and longer transcript level increases, suggest that the immune response is focused at the site of infection rather than systemically during staphylococcal SSTI.

Table 2 indicates that 49 genes with the greatest increases in transcript levels during the systemic response (comparing non-infected ears from challenged mice to naïve mice) are of unknown function. Of the characterized genes, 31 encode proteins that are involved in metabolism, cell proliferation, and regulation, and 20 in epithelial cell and/or hair formation. However, unlike in the local response, where many of the top genes had consistently increased transcript levels throughout the infection, most of the systemically up-regulated genes showed significant differential expression in the challenged mice compared to naïve, control mice at only one time point. Like with the local response, this trend was reflected in the entire list of systemically up-regulated genes (S1 Table and Fig 1C). We also saw that some genes had among the highest LFC at one time point, but were significantly down-regulated at another (indicated with italics, Table 2). These observations suggest that genetic changes happening systemically are more transient and that change occurs more quickly than at the site of infection, where genes with increased expression levels generally appear to exhibit more stable expression over time.

Table 3 contains the 50 genes with the greatest decreases in transcript levels at the site of infection, comparing infected ears to the uninfected ears from the same challenged mice. Again, many of these genes encode proteins with unknown function; those that have a known function appear important for cellular proliferation, differentiation, and metabolism. Only five of the genes in Table 3 encode proteins with an immune function. *Fcer2a*, which encodes an Fc receptor for IgE, has consistently decreased transcript levels at Days 1, 4, and 7, and *Serpinb1c* (which encodes a neutrophil proteinase inhibitor), is down-regulated starting at Day 4 post-challenge. Two genes that encode proteins important for binding to bacterial products (*Bpifb2* and *Lyg2*, which encode proteins that bind LPS and peptidoglycan, respectively) have significantly decreased transcript levels at Day 7. Forty-six of the genes that have the greatest decreases in transcript levels at the site of infection encode proteins that are involved in metabolism/cell proliferation/regulation, with the majority of these genes demonstrating transcript decreases early during infection (Day 1 or Day 4 post-challenge). We also found that, by day 7, 16 of the genes with the greatest decreases in transcript levels in response to local infection encode proteins that are involved in epidermal formation. However, the majority of genes with the greatest decreases in transcript level at each time point encode proteins of unknown function, with 62 of the top genes falling into this category. Unlike the genes that had significantly increased transcript levels, where a high proportion of genes had transcript increases at more than one time point, genes with significant decreases in transcript levels were mostly not shared over multiple times, which was observed both with the genes with greatest decrease in transcription (shown in Table 3) and in the entire list of genes with significant transcript decreases at the site of infection (S1 Table and Fig 1B). Out of 769 genes that had significant decreases in transcript levels in infected ears compared to non-infected ears from challenged mice, only 126 were shared over multiple time points, suggesting that the decrease in transcript levels of locally-expressed genes in our SSTI model is also more transient than transcript increases.

Table 4 contains all of the genes that had significant decreases in transcript levels systemically as there were fewer than 50 for each time point. Twelve of these genes encode proteins with an immune function. Ten of these twelve are decreased only at Day 7 post-challenge, and of these, six are involved in the adaptive response (*Ighg2c* and *Igkc*, which encode the gamma and kappa chains of immunoglobulin; *Pax5*, which encodes a protein involved in B cell differentiation [19]; *Ms4a1*, which encodes CD20, a regulator of B cell activation and proliferation [20], *Cd22*, which encodes a protein that functions in B cell signaling [21], and *Cd99*, which encodes a protein that plays a role in T cell migration into inflamed skin [22]). The majority of genes whose transcripts were decreased in non-infected ears from challenged mice compared to ears from naïve mice encode proteins that have functions in cellular metabolism, proliferation, or regulation, or lack a known function. Several genes that exhibited significant transcript decreases at one time point showed significant transcript increases at another (highlighted in italics in Table 4). Because the RNA preps from challenged mice used for this analysis are the same ones used in other analyses (the non-infected ear RNA used here as the test RNA is the same RNA used as the control RNA for examining at the local response) where we see very consistent expression of genes over time, we do not believe these temporal differences in gene expression are aberrant. The LFC values for systemic gene expression would also be affected by the number of reads in ears from naïve mice in these comparisons. However, because we used matched control mice that were kept in the same conditions and sacrificed at the same time as the challenged mice, we feel that any changes in gene expression due to environmental factors will be similar among all mice within a time point cohort. Therefore, we hypothesize the genes transcribed during the systemic response may have a transient expression pattern with rapid increases and decreases in transcript levels as the infection progresses.

**Fig 2:**
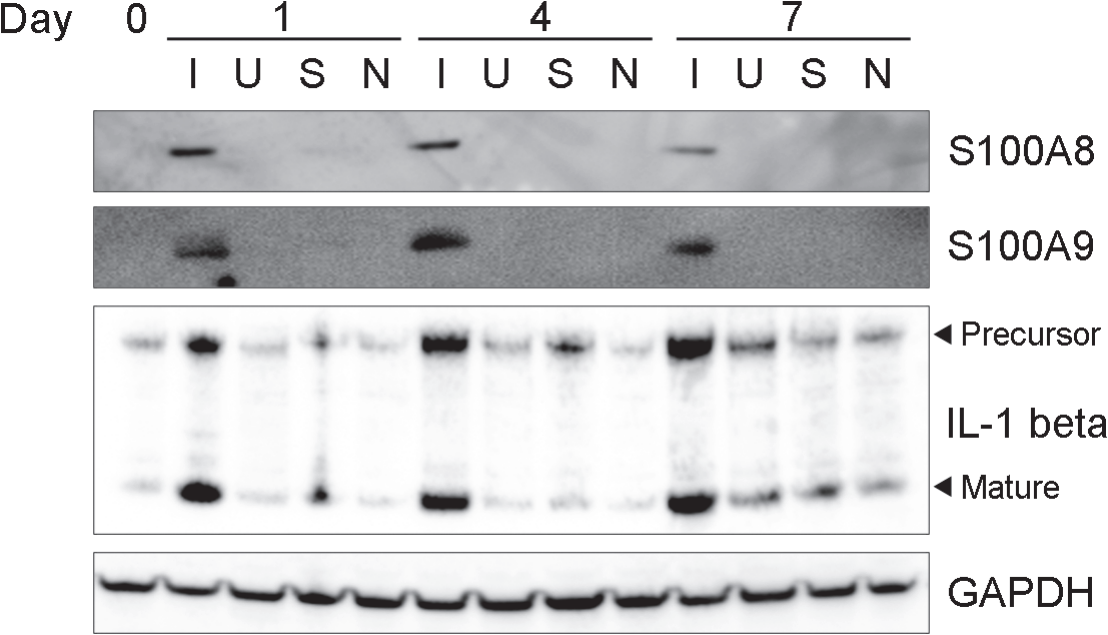
Protein expression for selected genes demonstrating locally increased transcripts. Western blots on ear lysates from infected ears (I), uninfected ears from the same challenged mice (U), sham-infected mice that were pricked with sterile PBS (S), and naïve, uninfected mice (N) using antibodies specific for S100A8, S100A9, IL-1β, and GAPDH at days 1, 4, and 7 post-challenge are shown. The precursor and mature forms of IL-1β are indicated.

In order to evaluate whether protein production mirrors gene expression, we performed Western blotting on homogenized ear lysates using antibodies specific for three proteins encoded by genes that were increased locally during infection. Western blots indicated that protein production for IL-1β, S100A8, and S100A9 is significantly increased upon infection (Fig 2, lanes denoted by “I”), which confirms the RNA-seq data for the genes encoding these proteins and further validates the RNA-seq data as a whole.

### Upstream Regulator Analysis of murine gene expression during SSTI

In order to funnel down the transcriptomic data, we next used the Upstream Regulator Analytic (URA) tool from the Ingenuity Pathway Analysis software package (www.ingenuity.com) to identify potential pathways that may be activated or repressed during *S*. *aureus* SSTI in the epicutaneous challenge model. This analysis software compares a curated database of genes and pathways to experimentally obtained transcriptomic data. It then uses statistical analysis to determine what percent of the genes within a pathway are differentially expressed in the data, and hypothesizes whether that pathway is activated or inhibited. If a high proportion of genes within a pathway are expressed in a manner that the database indicates signifies the pathway is functioning, URA will specify that the pathway is activated. Conversely, if a significant proportion of genes within a pathway are expressed in a manner opposite to what would be expected if the pathway is active, URA will indicate that the pathway is likely inhibited. Therefore, while URA is hypothetical, the analysis provides evidence-based suggestions of pathways that may be activated or inhibited based on transcriptomic data. It will also highlight activated or inhibited pathways that have upstream regulators that are not differentially expressed. These two factors allow for a global view of the transcriptomic data and for investigation into genes as pathway regulators that could otherwise be missed.

On Day 1 post-challenge, URA of the RNA-seq data for the local response (comparing infected ears to non-infected ears from the same challenged mice) indicated that 153 regulatory pathways were potentially activated at the site of infection. However, at this same time point, URA of the non-infected ears from challenged mice compared to naïve mice (systemic response) showed no activated pathways. At Day 4 post-challenge, there were 133 potentially activated pathways at the site of infection (local response), whereas URA of the systemic response showed only two activated pathways. At Day 7 post-challenge, URA of the RNA-seq data for the infected vs uninfected ears from the same mice (local response) showed indicated 127 possibly activated pathways, and URA of the RNA-seq data from the non-infected ears from challenged mice vs naïve mice (systemic response) showed 10 potentially activated pathways.

When evaluating inhibition of regulatory pathways, URA indicated 29 potentially inhibited pathways at the site of infection Day 1 post-challenge. On Day 4, 24 pathways were inhibited at the site of infection. When comparing uninfected ears from challenged mice and naïve mice to evaluate the systemic response, no pathways were inhibited on either Day 1 or Day 4. On Day 7 post-challenge, 23 pathways were potentially inhibited when analyzing the RNA-seq data from infected vs uninfected ears from challenged mice (local response). Systemically, three pathways were possibly inhibited when examining RNA-seq data from uninfected ears from challenged mice compared to naïve mice.

### Evaluation of upstream regulator activation during the course of SSTI

We next examined the URA analyses to categorize the types of pathways that were indicated as potentially activated during SSTI, both at the site of infection and at distal skin sites. For simplification, we are presenting only the top activated and inhibited pathways here (Tables 5 and 6; full URA data are available in S2–S7 Tables). In order to choose these top pathways, we sorted the URA data based on overlap *p* value, with the most significant *p* values listed first (signifying pathways that have the most significant overlap with pathways listed in the IPA Knowledge Base). We then further sorted for top pathways by choosing the most significant pathways (based on *p* value) that had an Activation Z score ≥ 2 (for activated pathways) or ≤ -2 (for inhibition). When more than 10 pathways met these criteria, we chose the 10 with the most significant *p* values.

**Table 5:** Top Activated Upstream Regulators^1^.

Upstream Regulator (Pathway)	Category	Activation Z score^2^	P value of overlap^3^
***Day 1 Local Response***			
TNF	cytokine	9.473	2.55E-55
NFkB (complex)	complex	7.035	3.92E-35
IFNG	cytokine	7.25	4.04E-34
IL-1β	cytokine	7.322	4.53E-34
RELA	transcription regulator	4.806	2.55E-28
IL-1α	cytokine	6.22	3.05E-27
STAT3	transcription regulator	2.822	3.75E-26
TREM1	transmembrane receptor	3.535	7.68E-26
JUN	transcription regulator	3.249	1.21E-21
TCR	complex	3.782	7.82E-21
***Day 1 Systemic Response***			
No activated upstream regulators			
***Day 4 Local Response***			
TNF	cytokine	8.697	1.48E-50
IFNγ	cytokine	6.83	5.39E-43
NFkB (complex)	complex	7.063	8.49E-37
IL-1β	cytokine	6.43	6.03E-35
IL-1α	cytokine	6.069	2.91E-30
TCR	complex	3.741	3.19E-24
TREM1	transmembrane receptor	3.493	5.09E-23
RELA	transcription regulator	4.386	7.45E-23
JUN	transcription regulator	3.223	1.55E-22
IL-27	cytokine	2.403	5.84E-21
***Day 4 Systemic Response***			
IL-1α	cytokine	2.184	8.80E-08
TNF	cytokine	2.572	1.66E-07
***Day 7 Local Response***			
IFNγ	cytokine	7.934	1.78E-52
TNF	cytokine	8.592	1.11E-44
NFkB (complex)	complex	6.327	1.68E-31
TCR	complex	3.319	1.38E-29
IL-1β	cytokine	5.528	1.26E-27
TGM2	enzyme	7.107	9.06E-26
IL-27	cytokine	2.837	3.28E-25
IL-12 (complex)	complex	3.029	3.98E-24
STAT3	transcription regulator	2.78	6.94E-24
IL-21	cytokine	3.548	9.54E-24
***Day 7 Systemic Response***			
IL-17A	cytokine	2.376	3.82E-11
TNF	cytokine	2.617	2.88E-10
TLR7	transmembrane receptor	2.399	7.15E-10
IL-1β	cytokine	2.31	1.28E-09
CAMP	other	2.219	2.49E-09
NFkB (complex)	complex	2.606	2.96E-09
RELA	transcription regulator	2.343	3.98E-09
IL-1α	cytokine	2.416	5.87E-09
SELPLG	other	2	2.46E-07
ERK1/2	group	2.19	1.45E-06

^1^Top upstream regulators selected based on p value of overlap and activation Z score. Data are ordered by p value of overlap. If more than 10 pathways were indicated as activated for a condition, the top 10 regulators were chosen as the 10 most significant regulators (based on p value) that had a Z score ≥ 2.

^2^Z score infers the activation states of predicted regulators based on expression of the downstream genes within the pathway; a score ≥ 2 indicates activation.

^3^P value of overlap evaluates whether there is a statistically significant overlap between differentially expressed genes and the genes that are regulated by the upstream regulator.

**Table 6:** Top Inhibited Upstream Regulators^1^.

Upstream Regulator (Pathway)	Category	Activation Z score^2^	P value of overlap^3^
*Day 1 Local Response*			
JAG2	growth factor	-4.32	3.98E-15
MAP3K7	kinase	-2.316	2.20E-14
IgG	complex	-2.187	1.97E-13
CD3	complex	-4.555	3.55E-13
TAB1	enzyme	-3.85	6.56E-13
miR-155-5p (miRNAs w/seed UAAUGCU)	mature microRNA	-3.394	9.54E-11
IL1-RN	cytokine	-4.776	1.35E-10
TRAF3	enzyme	-2.345	4.52E-10
CD28	transmembrane receptor	-2.823	4.99E-10
MAPK1	kinase	-4.137	6.48E-10
*Day 1 Systemic Response*			
No inhibited upstream regulators			
*Day 4 Local Response*			
CD3	complex	-4.135	4.18E-17
IL1-RN	cytokine	-4.981	1.43E-15
JAG2	growth factor	-3.961	1.57E-13
CD28	transmembrane receptor	-3.064	4.94E-13
miR-155-5p (miRNAs w/seed UAAUGCU)	mature microRNA	-3.406	8.37E-13
TAB1	enzyme	-3.582	5.26E-12
miR-146a-5p (and other miRNAs w/seed GAGAACU)	mature microRNA	-3.494	1.52E-10
IL-37	cytokine	-2.408	1.22E-07
MAPK1	kinase	-3.297	2.02E-07
SOCS1	other	-2.959	2.06E-07
*Day 4 Systemic Response*			
No inhibited upstream regulators			
*Day 7 Local Response*			
CD3	complex	-4.313	2.37E-19
IL1-RN	cytokine	-4.902	4.48E-19
MAPK1	kinase	-4.783	1.18E-16
TAB1	enzyme	-3.359	3.72E-15
miR-155-5p (miRNAs w/seed UAAUGCU)	mature microRNA	-3.159	2.59E-14
CD28	transmembrane receptor	-3.421	3.47E-14
JAG2	growth factor	-3.221	2.31E-12
SOCS3	phosphatase	-2.376	2.38E-10
miR-146a-5p (and other miRNAs w/seed GAGAACU)	mature microRNA	-3.357	1.22E-09
SOCS1	other	-3.124	1.43E-08
*Day 7 Systemic Response*			
miR-155-5p (miRNAs w/seed UAAUGCU)	mature microRNA	-2.607	5.40E-14
JAG2	growth factor	-2.216	2.49E-09
IL-13	cytokine	-2.200	2.12E-05

^1^Top upstream regulators selected based on p value of overlap and activation Z score. Data were ordered by p value of overlap. If more than 10 pathways were indicated as inhibited for a condition, the top 10 regulators were chosen as the 10 most significant regulators (based on p value) that had a Z score ≤ -2.

^2^Z score infers the activation states of predicted regulators based on expression of the downstream genes within the pathway; A score ≤ -2 indicates inhibition.

^3^P value of overlap evaluates whether there is a statistically significant overlap between differentially expressed genes and the genes that are regulated by the upstream regulator.

The top activated pathways are listed in Table 5. The majority of top pathways activated in the local response (comparing the infected ears to non-infected ears from challenged mice) were shared across all three tested time points and several were activated systemically by Day 7 post-challenge. The TNF pathway is activated locally on Days 1, 4, and 7, and systemically (comparing non-infected ears from challenged mice to naïve mice) on Days 4 and 7. The NFκB and IL-1β pathways are activated locally at all three time-points, and systemically on Day 7. The IL-1α pathway was among the top activated pathways locally on Day 1, both locally and systemically on Day 4, and systemically on Day 7. It was also activated locally on Day 7, though it was not a top pathway under this condition (Z score 5.045, *P* value 1.04 x10^-19^; S6 Table). The RELA pathway was a top activated pathway locally at Days 1 and 4, and systemically on Day 7. This pathway was activated locally at Day 7 also, but again was not among the top pathways (Z score 3.822, *P* value 3.27 x 10^–23^; S6 Table). Other top pathways were activated only locally at the site of infection, including IFNγ, TCR, STAT3 (not on the top pathway list at Day 4; Z score 2.459, *P* value 1.72 x 10^–19^; S4 Table), TREM1 (not on the top pathway list at day 7; Z score 2.242, *P* value 8.72 x 10^–21^; S6 Table), JUN (not on the top pathway list at day 7; Z score 2.910, *P* value 9.77 x 10^–16^; S6 Table), and IL-27 (not on the day 1 top activated pathway list; Z score 2.168, *P* value 3.6 x 10^–16^; S2 Table). The top activated pathways at all three tested time points are all involved in the immune response. In particular, these pathways suggest the importance of the TH1 (IFNγ, TREM1, IL-27), TH17 (STAT3, IL-1β, IL-17A), and overall pro-inflammatory responses (NFκB, IL-1α, RELA, JUN, ERK1/2, SELPLG).

Pathway inhibition was similar across all three time points as well, with 24 out of 43 inhibited pathways shared at Days 1, 4, and 7 (S2–S7 Tables). As with the activated pathways, we also listed the top inhibited pathways in Table 6. Of these top pathways, two (JAG2 and Mir-155-5p) were inhibited locally at all three tested time points, and systemically at Day 7, and five were among the top locally inhibited pathways at all three time points but unaffected systemically (CD3, IL-1RN, TAB1, CD28, and MAPK1). Two pathways were on the top inhibited pathways lists at Days 4 and 7, and were also inhibited on Day 1, but did not make the top list for this time point (Mir-146a-5p, Z score -3.075, *P* value 9.5 x 10^–7^; SOCS1, Z score -2.959, *P* value 5.41 x 10^–6^; S2 Table). IL-37, which was a top locally inhibited pathway on Day 7, was also inhibited on Days 1 (Z score -2.408, *P* value 8.77 x 10^–7^; S2 Table) and 4 (Z score -2.408, *P* value 1.22 x 10^–7^; S4 Table) but was not a top inhibited gene at these times. The SOCS3 pathway was a top locally inhibited pathway at Day 7, and was also locally inhibited at Day 4 (Z score -2.619, P value 2.21 x 10^–7^; S4 Table) but was not inhibited at day 1. These data further support the similarity of the response over time during *S*. *aureus* SSTI. As with the top activated pathways, the top inhibited pathways all function in the immune response. These pathways generally appear to be involved in the anti-inflammatory response (e.g., SOCS1 and SOCS3, which work to suppress cytokine production, and IL-37, which inhibits innate immunity [23]). Inhibition of these anti-inflammatory pathways may augment a pro-inflammatory response. We also found that the CD3 and CD28 pathways, both critical to T cell receptor signaling [24], were consistently inhibited at all three time points.

Though we did see a higher level of variability in the RNA-seq data in terms of differential expression of individual genes at the tested time points (refer to Tables 1–4, which demonstrate that a number of the genes that are highly differentially expressed at one time are not differentially expressed at other times), the URA suggests that global pathway activation and repression is similar over the course of infection.

## Discussion

In order to better understand the effects of staphylococcal infection on the host, we performed a comprehensive genetic analysis of the mouse transcriptome using high-throughput sequencing of cDNA (RNA-Seq) prepared from RNA enriched from mouse ears using an epicutaneous infection model [13]. In this study, we chose to compare infected ears to uninfected ears from the same challenged mice in order to evaluate the local response at the site of infection. We also compared cDNA from the uninfected ears from challenged mice to cDNA from ears of naïve mice in order to determine differential gene expression systemically over the time course of infection. Through these analyses, we determined that a large number of genes are differentially expressed at the site of infection both early and late during SSTI, while a smaller number of genes are affected systemically. When we categorized these differentially expressed genes into pathways using the IPA Upstream Regulator Analytic (URA), we found that all of the pathways that showed the highest level of significance both locally and systemically were involved in the immune response.

URA bundles transcriptomic data together into potentially activated or inhibited pathways, which can give hints as to what is happening within the cellular environment to lead to the observed expression data results. URA can predict activation or inhibition of pathways whether or not the gene encoding the upstream regulator itself is differentially expressed, providing added benefit over examining gene expression data alone. The majority of the predicted activated pathways, in fact, did not have a differentially expressed upstream regulator (S2–S7 Tables). For example, both the IL-12 and IL-18 pathways were predicted to be activated at every time point (S2–S7 Tables), and while *Il-12a* and *Il-12b* show significantly increased transcript levels, the *Il-18* gene itself was not differentially expressed (S1 Table). If one were only examining the gene expression data, the potential importance of the IL-18 pathway could be missed. These analyses broaden avenues of future study of staphylococcal SSTI using a rational, statistically-based categorization of gene expression data. However, it is important to note that the URA is theoretical, and follow-up experiments are necessary to confirm the activation or inhibition of these pathways.

The immune-associated genes that demonstrated the highest differential expression at the site of infection (Table 1) suggest a significant local inflammatory response. Our data largely agree with the microarray analysis performed by Cho and colleagues [9,11]; all 20 top up-regulated genes at four hours post-infection in the previous dataset had significantly increased transcript levels in our SSTI model at Day 1 post-infection. The activation of the IL-1β pathway at all tested time points in our data, demonstrated through URA, also supports Cho *et al*.’s contention that this cytokine plays an important role in SSTI. Our URA predicts that the IL-1α pathway was also activated at the site of infection at all tested time points; this pathway was activated systemically both at Day 4 and Day 7. Several pathways that are important for the TH17 response were activated at the tested time points, including the TNF, IL-6, TGF-β1, IL-21, and GM-CSF pathways. Cho and colleagues also demonstrated the importance of IL-17 in resolution of *S*. *aureus* skin infection [11]. *Il-17a* was also one of the top locally up-regulated genes Day 1 post-challenge in our model, and was IL-17 was activated globally as a pathway regulator (meaning that a significant number of downstream genes were differentially expressed) at all three time points.

We feel that the high degree of similarity between our Day 1 data and Cho *et al*.’s four hour post-infection data [9] validate the results we obtained through RNA-seq. Future studies will follow up on the biological significance of several genes that we have found to be differentially expressed in our model.

Our data expand on Cho and colleagues’ analyses through testing more time points and through use of a more sensitive method of studying differential gene expression. We also evaluated both local and systemic responses to *S*. *aureus* SSTI instead of focusing only on the response at the site of infection. In order to best elucidate protective immune responses, we believe it is important to fully understand gene expression both at the site of infection and at distal sites.

While a significant proportion of differentially expressed genes were shared between Days 1, 4, and 7 (Fig 1), we also found many genes that are differentially expressed early and not late, or vice versa. For example, *Saa2*, which encodes an acute phase protein, has significantly increased transcript levels at the site of infection on Days 1 and 4, but is not differentially expressed at Day 7. SAA2 is important in the initial response to infection and its expression is activated by pro-inflammatory cytokines [25]. *Csf2*, which encodes a cytokine involved in granulocyte production, and the gene encoding Ficolin B, a pattern recognition receptor [26], both demonstrate significant transcript increases only at Day 1 at the site of infection. Conversely, the gene encoding granyzme K, a cytolytic granule found in CTLs and NK cells [27], had signfiicant increases in transcript levels at the site of infection only at Day 7. These genes’ expression profiles give a sense of how the host response is changing over time.

We also found a large number of uncharacterized genes that were differentially expressed early during infection but not late, or vice versa. These genes could be potentially interesting as unknown mediators of any number of pathways, host responses, cellular metabolism, etc. Because SSTI is self-limiting [13], we hypothesize that that the genes that are differentially expressed later but not early are potentially most important for direct resolution of the infection, and may provide insight into the response that leads to clearance by 14 days post-challenge. However, those genes that are expressed only early in the infection might be important for helping to stimulate later responses, or for holding bacterial levels in check, allowing for future resolution.

Through examining gene expression over time, we also were able to demonstrate that activation of systemic immune responses lags behind that of the local response. Of the immune genes with the most significantly systemically increased transcript levels only on Day 7 (see Table 2), all except for two (*Il-20* and *Defb6*) had significant increases in transcripts locally by one day after infection (Table 1 and S1 Table). The kinetics of infection, along with the patterns of gene expression, may help aid in dissection of temporal changes that lead to clearance.

Our gene expression data suggest that a TH1 response is occurring during staphylococcal SSTI. *Ifnγ* had significant increases in transcript levels at the site of infection on Days 4 and 7 (LFC = 4.15 and 4.43, respectively; S1 Table), and its pathway was also predicted through URA as activated locally at Days 1, 4, and 7 after challenge because a large number of genes affected by IFNγ demonstrated expression patterns indicative of pathway activation (S2, S4 and S6 Tables). This cytokine is important for a variety of immune functions, including activating innate immune cells and skewing the adaptive response toward TH1 [28]. IL-12 and IL-18 synergistically amplify IFNγ production [29], increasing the TH1 response. Both genes encoding IL-12 had significantly increased transcript levels at the site of infection in our model (*Il-12a* Day 1 LFC 2.84; Day 4 LFC 2.00; *Il-12b* Day 4 LFC 2.23, Day 7 LFC 4.29; S1 Table). We also saw significant local transcript increases of genes encoding the IFNγ-inducible TH1 chemokines CXCL9 (Table 1), CXCL10 (LFC 4.21, 4.59, and 4.87, respectively), and CXCL11 (LFC 2.09, 3.75, and 5.21, respectively) at Days 1, 4, and 7 [30]. Whether this response is beneficial is debatable, as Montgomery and colleagues demonstrated that the TH1/IFNγ pathway prevented protective immunity in their model of SSTI [31], and Nippe *et al*. demonstrated in a different subcutaneous infection model that mice skewed toward a TH1 response exhibited increased swelling at the infection site and higher bacterial loads by one week post-challenge [32]. The pathways controlled by IL-4, the hallmark cytokine of a TH2 response [28], as well as TH2 cytokine IL-13, were not activated in our model. In fact, the IL-13 pathway is predicted to be inhibited systemically by Day 7 post-challenge (S7 Table). These results are suggestive that, at least in our epicutaneous model of SSTI, the TH1 response may be augmented over the TH2 response. Previous work demonstrated that anti-alpha toxin antibody levels can correlate at least partially to protection in this model [33], but it remains unclear what mechanism of protection is most important in natural infection. The TH1 response augments the production of complement-fixing and opsonizing antibodies [34], which could be important in the response to *S*. *aureus*. Further studies will be required to dissect TH1/TH2 responses in the epicutaneous SSTI model.

Besides the mediators discussed above, a substantial number of other genes encoding proteins that are important for the innate response had significantly increased transcript levels, or their pathways were predicted as activated based on the transcriptional data, in our SSTI model. These include a variety of other cytokines and chemokines, factors important for leukocyte adhesion, components of the complement cascade, proteases found in neutrophil granules, and mast cell proteases. Since the infection is self-limiting, remains localized, and clears by day 14, it is likely that the innate response plays an important role in containing *S*. *aureus* SSTI. Neutrophils have been implicated for their importance in controlling these infections [8,9,13]. Our results suggest that other components of the innate response, such as macrophages stimulated through the TH1 response, may also play significant roles, and future work will begin to dissect these mechanisms.

Keratinocytes are now recognized for their importance in regulating the immune response in the skin [35]. These cells produce cytokines and chemokines as well as express cytokine and chemokine receptors. A considerable number of keratin genes had increased transcript levels both locally and systemically during SSTI, especially early in the infection (S1 Table). Besides their importance as major cytoskeletal proteins in the epithelia [36], some keratins have been identified as important regulators of the immune response. While keratin genes have been examined for their roles in inflammatory diseases such epidermolytic icthyosis [37], their role in the response to staphylococcal pathogenesis has to this point remained undescribed. These genes may provide novel means of targeting these infections. The gene encoding KRT17, which promotes epithelial cell proliferation as well as a TH1/TH17 response [38], had significantly increased transcript levels at the site of infection at Days 1 and 4 post-challenge (LFC 1.76 and 1.39, respectively), and K*rt16* was highly expressed at the site of infection at all three time points, with LFCs greater than 5. KRT16 was identified for its importance in innate immune regulation during epithelial infection, where it may provide an important checkpoint in the pro-inflammatory feedback loop [39]. KRT1 is involved in negative regulation of the pro-inflammatory response in the epithelia [40], and *Krt1 was* significantly up-regulated at Days 4 and 7 at the site of infection in our model (LFC 1.36 and 1.77, respectively). Roth and colleagues established that *Krt1* ^*-/-*^ mice have markedly higher levels of the pro-inflammatory cytokine IL-33 [40]. We noted increased transcript levels of *Il-33* in infected ears at Day 4 (LFC 1.38, S1 Table). However, by Day 7, *Il-33* transcripts are no longer increased, while *Krt1* transcripts remain increased. This suggests that KRT1 may play a role in dampening inflammation during *S*. *aureus* SSTI, possibly by modulating IL-33 levels. By Day 7, 50 keratin-associated genes had significantly decreased transcript levels at the site of infection (S1 Table). We hypothesize that keratins may be part of the immune balance in the host during *S*. *aureus* SSTI, through rapid early increases in keratin gene expression, perhaps to increase cell number/epithelial thickness, as well as to augment and control the innate immune response; as the infection continues, tissue damage worsens and the adaptive response begins to take over. At this point, the keratin genes are down-regulated. KRT8 has been postulated as a potential binding site for *S*. *aureus* adhesin ClfB [41]. Interestingly, the *Krt8* gene is significantly down-regulated at the site of infection in our model at Day 4 (LFC -1.31, S1 Table). This lowered expression may help the host to decrease bacterial adherence in an effort to limit infection. Overall, the keratins are a largely uninvestigated area of the *S*. *aureus*/host interaction that deserves greater attention.

While the current literature studying responses to *S*. *aureus* SSTI have focused on inflammatory responses, our data suggest that genes that help to keep inflammation in check may also be important in the host response. A number of differentially expressed genes at the site of infection encode proteins with immune modulating functions. These include *Nlrp12* (Table 1) [42], *Nlrp6* (significantly increased at the site of infection on Days 1 and 4; S1 Table) [43], *Irg1* (Table 1) [44], *Olfm4* (Table 1) [45], *Soscs3* (locally increased transcript levels on Days 1, 4, and 7; S1 Table) [46], zinc finger protein *Zc3h12a* (locally increased transcript levels on days 1 and 4; S1 Table) [47], and several *Cd300* family members (both increased and decreased transcript levels, S1 Table) [48]. We also note that a significant proportion of inhibited pathways are involved in attenuation of inflammation (Table 6 and S2–S7 Tables). We hypothesize that the proteins encoded by these genes may help prevent dysregulated inflammation that could exacerbate skin damage; however, it is likely that a number of anti-inflammatory pathways must also be inhibited in order to obtain a level of inflammation necessary to clear *S*. *aureus*. This immune balance could be critical for generation of a successful response to the pathogen. Further research into these pathways is required to determine their actual role in the response to SSTI.

In summary, we present a comprehensive transcriptomic analysis of the time course of murine gene expression for up to one week after *S*. *aureus* challenge in an epicutaneous skin infection model. We examined differential gene expression both at the site of infection as well as systemically at three time points by comparing infected ears to uninfected ears from the same challenged mice, or comparing uninfected ears from challenged mice to naïve mice. We also used computational analysis of the transcriptomic data to generate predictions on potentially activated and inhibited pathways during both the local and systemic response to infection over time. Through these evaluations we found that the systemic response lags behind the local response in terms of pathway activation and inhibition. We determined that a majority of the differentially expressed immune response genes are critical for the innate, TH17, and TH1 responses. We also found that a large number of keratin-associated genes are differentially expressed over time, which could give insight into tissue damage, remodeling, and the potential immune function of these genes. The high level of differential gene expression, both at the site of infection and systemically, provides a great deal of potential avenues for further study into the response to this infection. RNA-seq does not provide information as to which gene(s) are responsible for resolution of infection in our model; instead, the data generated from this work allow us to develop testable hypotheses that may further our knowledge in this area. These data may help us understand how the host can contain the infection in this self-limiting model. Further study using other animal models of SSTI, including humanized mice, will help to determine if the differences in gene expression we elucidated may be applicable to human diseases.

## Supporting Information

S1 Table Log fold change (Log_2_) for all RNA-seq data. (XLS)

S2 Table URA of RNA-seq data from local response, Day 1. The data used for this analysis compared infected ears to non-infected ears from challenged mice.
(XLS)

S3 Table URA of RNA-seq data from systemic response, Day 1. The data used for this analysis compared non-infected ears from challenged mice compared to naïve mice.
(XLS)

S4 Table URA of RNA-seq data for local response, Day 4. The data used for this analysis compared infected ears to non-infected ears from challenged mice.
(XLS)

S5 Table URA of RNA-seq data for systemic response, Day 4. The data used for this analysis compared non-infected ears from challenged mice compared to naïve mice.
(XLS)

S6 Table URA of RNA-seq data for local response, Day 7. The data used for this analysis compared infected ears to non-infected ears from challenged mice.
(XLS)

S7 Table URA of RNA-seq data for systemic response, Day 7. The data used for this analysis compared non-infected ears from challenged mice compared to naïve mice.
(XLS)
